# A Review of Vision-Based Multi-Task Perception Research Methods for Autonomous Vehicles

**DOI:** 10.3390/s25082611

**Published:** 2025-04-20

**Authors:** Hai Wang, Jiayi Li, Haoran Dong

**Affiliations:** The School of Automotive and Traffic Engineering, Jiangsu University, Zhenjiang 212013, China; ruth_jiayi_li@163.com (J.L.); dhr710@outlook.com (H.D.)

**Keywords:** multi-task learning, autonomous driving, detection, drivable area segmentation, deep learning

## Abstract

Multi-task perception technology for autonomous driving significantly improves the ability of autonomous vehicles to understand complex traffic environments by integrating multiple perception tasks, such as traffic object detection, drivable area segmentation, and lane detection. The collaborative processing of these tasks not only improves the overall performance of the perception system but also enhances the robustness and real-time performance of the system. In this paper, we review the research progress in the field of vision-based multi-task perception for autonomous driving and introduce the methods of traffic object detection, drivable area segmentation, and lane detection in detail. Moreover, we discuss the definition, role, and classification of multi-task learning. In addition, we analyze the design of classical network architectures and loss functions for multi-task perception, introduce commonly used datasets and evaluation metrics, and discuss the current challenges and development prospects of multi-task perception. By analyzing these contents, this paper aims to provide a comprehensive reference framework for researchers in the field of autonomous driving and encourage more research work on multi-task perception for autonomous driving.

## 1. Introduction

With the rapid development of intelligent and connected vehicles and intelligent transportation systems [1,2,3], autonomous driving technology has become a research hotspot. Autonomous driving technology mainly includes three modules, namely, environment perception, decision-making and planning, and motion control. Among them, environment perception, as the primary link, provides accurate and reliable data support for the subsequent decision-making and planning and motion control modules, thus playing a crucial role in ensuring the normal driving of vehicles. Autonomous driving environment perception mainly involves the recognition of dynamic and static obstacles, as well as road surface information. Common tasks include traffic object detection, drivable area segmentation, and lane detection.

Traditional research methods usually focus on single-task perception, but autonomous driving scenarios require the simultaneous acquisition of various types of information on the road. Compared with designing a separate neural network for each task, multi-task learning can reduce the total number of parameters of the neural network, decrease the complexity of the network deployment, and reduce the occupation of hardware resources. Multi-task environment perception models usually combine the characteristics of multiple tasks to design a backbone network that meets the requirements. The parameters are shared among multiple branches, thus reducing the computational load. Therefore, for the environment perception tasks in autonomous driving scenarios, the study of multi-task perception models and their embedded deployment acceleration is of great significance for the implementation of autonomous driving applications and has become an urgent need for developers.

This paper systematically reviews the research results in the field of multi-task perception for autonomous driving, covering the core tasks of traffic object detection, drivable area segmentation, and lane detection; combs through the classification, classical architecture, and loss function design of multi-task learning; combines the commonly used datasets and evaluation metrics; and puts forward the challenges faced by the current technology and the future direction, which provides theoretical support and practice for the development of multi-task perception systems.

This paper is organized as follows: in Section 2, the research results in the field of multi-task perception for autonomous driving are reviewed, covering three core tasks: traffic object detection, drivable area segmentation, and lane detection. Then, in Section 3, the definition of multi-task learning, its role, and its classification are explored. Additionally, the classical network architecture for multi-task perception and the design of its loss function are analyzed to provide a clear framework for understanding the application of multi-task learning in the field of autonomous driving. Section 4 introduces commonly used datasets and evaluation metrics in the field of multi-task perception for autonomous driving. Section 5 presents our views on the current challenges and future directions of multi-task perception for autonomous driving.

## 2. Relevant Technical Background

### 2.1. Classification of Perception Tasks

Multi-task environment perception for autonomous driving is a multi-dimensional and complex problem involving the comprehensive understanding of dynamic traffic participants, static road elements and environmental features. Around the application of multi-task learning for autonomous driving perception, the core research content can be divided into the following three types of typical tasks.

Object detection tasks: these are represented by traffic object detection, focusing on identifying the locations and categories of dynamic objects such as vehicles, pedestrians, and cyclists;

Scene understanding tasks: these include drivable area segmentation and lane detection, aiming at constructing semantic representations of road geometry;

Complementary enhancement tasks: these cover complementary tasks such as traffic sign recognition, depth estimation, and target tracking to provide additional environment information.

In this paper, we focus on three major tasks, namely, traffic object detection, drivable area segmentation, and lane detection. The following section will specifically analyze the research methodology and research progress of these three tasks.

### 2.2. Traffic Object Detection

**Figure 1:**
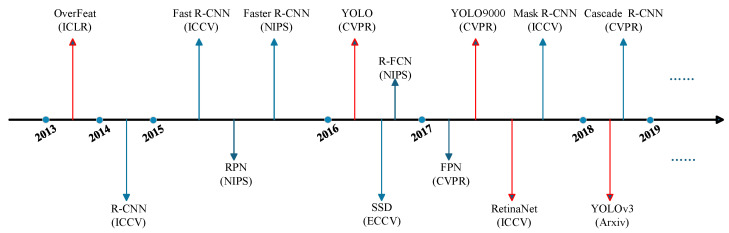
Common algorithms for object detection.

In recent years, with the booming of deep learning and computer vision [4,5,6,7], a large number of models have emerged in the field of object detection. Current object detectors are mainly classified into two-stage and one-stage detectors. Common detectors are presented in Figure 1 below, where the blue arrows point to the two-stage detector and the red arrows point to the one-stage detector. The two types of detectors have their own focuses in multi-task perception for autonomous driving. The two-stage detectors prioritize accuracy, while the one-stage detectors emphasize real time. The technical characteristics of the two have a direct impact on the architectural design of the multi-task model and the efficiency of the task collaboration.

#### 2.2.1. Two-Stage Object Detectors

Two-stage object detectors use a step-by-step strategy to accomplish the object detection task. First, region proposals are obtained by region proposal generation methods. Then, the classification of the objects within these regions is carried out via feature extraction, and the locations of these regions are refined with the help of the bounding box regression technique. The important first step is the generation of region proposals, which refers to an algorithm that automatically generates a sparse set of candidate regions in the image where target objects may be present. This process narrows down the processing for subsequent accurate classification and localization. The two-stage object detectors are represented by the R-CNN family of models, including R-CNN [8], Fast R-CNN [9], and Faster R-CNN [10]. In addition, network modules such as SPP [11] and RPN [10] are used for improvement.

R-CNN [8] uses Selective Search [12] to generate region proposals. This is a region generation method based on image features. It splits the image into different small regions based on the color, texture, boundary, and other features of the image; then merges these small regions to form region proposals that may contain the target; and resizes them to a 227 × 227 size. It then uses a CNN to extract features from each region proposal and obtain a 4096-dimensional feature vector. Finally, an SVM classifier is used to classify and localize the object. However, the resizing operation may cause the deformation of the object’s appearance, thereby degrading the learning performance of the model. Moreover, it necessitates a large number of CNN operations, leading to substantial redundant computations, which makes both the training and testing processes extremely time-consuming and difficult to optimize jointly with other tasks. SPP [11] employs a spatial pyramid pooling method that accepts input feature maps of arbitrary sizes, eliminating the need for resizing operations. It performs feature extraction on the entire image first and then obtains the features of each region proposal, resolving the issue of redundant convolutions through a single feature extraction step and accelerating the training process. However, it still relies on the Selective Search method for the region proposal generation. Fast R-CNN [9] integrates the advantages of SPP into R-CNN and proposes RoI pooling, which employs single-scale max pooling for the feature extraction and softmax for the classification. This architecture restructures R-CNN into a parallel framework to simultaneously handle the classification and regression. However, it still relies on the computationally intensive Selective Search method to generate the region proposals. In order to address this issue, Faster R-CNN [7] proposes an RPN network that can efficiently generate region proposals with a wide range of scales and aspect ratios by sliding a small window over the feature map and predicting the target scores and bounding box offsets at each window location, enabling end-to-end training for the first time and reducing the proposal generation time by more than 90%.

#### 2.2.2. One-Stage Object Detectors

Although two-stage object detectors are more accurate, they have the significant drawbacks of a long training time and a slow inference speed. As a result, more efficient end-to-end one-stage object detectors, which are more popular in embedded systems, have emerged. In contrast to the two-stage object detectors, one-stage object detectors omit the complex process of region proposal generation and cascade classification, predict the target categories and coordinates directly on the feature map, significantly improve the inference speed, and have become the first choice for real-time solutions in multi-task perception.

The most commonly used one-stage object detectors are the YOLO series and the SSD series. The core idea of YOLO [13] is to extract features from the input image through the backbone network, and then divide the obtained feature map into an S×S grid. If the center of an object falls within a certain grid, this grid is responsible for predicting the confidence, category, and coordinate position of the object. YOLO9000 [14] introduces the anchor box mechanism and adopts a deeper Darknet-19 network structure, which improves the detection accuracy and recall rate. YOLOv3 [15] introduces a multi-scale prediction mechanism. Using the feature pyramid network structure, it conducts object detection on feature maps of three different scales, enhancing the model’s ability to detect small objects. YOLOv4 [16] makes multiple innovations in the network structure. It adopts the Mish activation function, introduces a new loss function, and applies various data augmentation techniques, further improving the robustness of the object detection. YOLOv5 and YOLOv8 dramatically increase the speed while maintaining the detection accuracy through a lightweight backbone and adaptive anchor frame computation. YOLOv11 inherits from YOLOv8. It uses a streamlined design, making it suitable for various applications and easily adaptable to different hardware platforms from edge devices to cloud APIs. It has become an excellent choice for various object detection and tracking, instance segmentation, image classification, and pose estimation tasks. SSD [17] is a one-stage anchor-based object detector designed to address some limitations of YOLOv1. Different from YOLOv1, SSD does not require an additional region proposal process. Instead, it generates a set of multi-scale anchors with different aspect ratios in each grid cell to detect objects of different sizes.

Object detection algorithms are widely applied in multiple fields. They are not only suitable for the detection of traffic objects but also play a crucial role in the agricultural field, especially in the detection of flowers and fruits. For example, the improved YOLOv4 algorithm was used for apple recognition and localization [18]. Similarly, YOLOv8 was applied to locate strawberry picking points [19]. A lightweight model based on YOLOv4-Tiny was used for the detection and localization of potted flowers [20]. These studies provided technical support for the development of agricultural picking robots. To improve the efficiency of robots in picking apples in complex orchard environments, Ji et al. [21] proposed a method combining the improved YOLOv5 and a density clustering algorithm. By optimizing the object detection and picking path planning, the method significantly improved the picking efficiency and success rate. In addition, in the field of grape picking, researchers developed a fast picking point localization method based on multi-grape synchronous inference. The method achieved high-precision fruit recognition and picking point localization through the feature-enhanced model YOLOv4-SE [22].

### 2.3. Drivable Area Segmentation

In the era of deep learning, semantic segmentation has made significant progress, as has object detection. While object detection focuses on the objects in an image, classifying and localizing them by using bounding boxes, semantic segmentation classifies each pixel in an image to provide detailed and accurate information about the scene. Therefore, most of the tasks for drivable area detection use semantic segmentation to get pixel-level results for drivable areas.

In traditional machine vision, semantic segmentation mainly relies on some basic 2D attributes, such as shape, color, texture, and other feature information. Such methods are committed to enhancing the consistency of features within the same object while increasing the differentiation of features between different objects. Traditional methods can be divided into two categories: segmenting gray-scale images and segmenting color images. They are also often used in the field of agricultural engineering. Specifically, these methods include thresholding-based [23,24,25], region-based [26,27], edge-based [28,29], graph theory-based, clustering-based [30,31], and energy functional-based methods.

**Figure 2:**
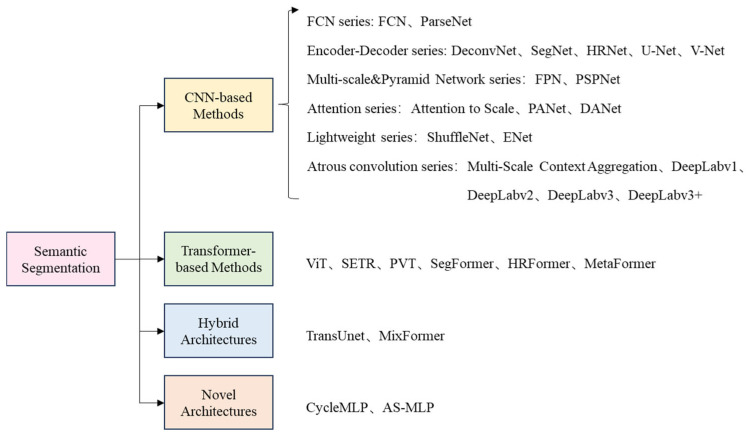
Common algorithms for semantic segmentation.

In recent years, with the rapid progress of neural networks, image semantic segmentation methods based on deep learning have moved into a new stage. The architectural designs of semantic segmentation models have become increasingly diverse. According to different segmentation method systems, they can be classified into four categories: CNN-based, Transformer-based, hybrid architectures, and novel architectures. The specific algorithms are shown in Figure 2 below.

In precision agriculture, both CNN-based and Transformer-based segmentation methods have been applied. Peng et al. [32] studied three semantic segmentation networks, namely, FCN [33], U-Net [34], and DeepLabv3+ [35], for the segmentation of grape clusters of different varieties. They explored the performance differences among different networks, the impact of input representation, the effect of image enhancement, and the influence of the distance between the object and the camera on the segmentation performance. To improve the efficiency of intelligent apple-picking robots, Tang et al. [36] proposed an instance segmentation method based on RGB-D images and an improved SOLOv2. This method uses EfficientNetV2 as the feature extraction network and introduces a lightweight spatial attention module to enhance the segmentation accuracy in cases of occlusion and overlap. Some other studies have explored the use of Transformer. In terms of crop disease identification, Zhu et al. [37] proposed a new model. By introducing the Transformer encoder to extract global features and using Centerloss to optimize the loss function, the discrimination between different diseases was improved. For apple quality grading, Ji et al. [38] developed an improved model based on YOLOv5s, specifically introducing the Swin Transformer to enhance the grading accuracy. These studies indicate that both CNN-based and Transformer-based semantic segmentation methods have demonstrated significant advantages in the field of agricultural image processing. They not only expand the application scope of semantic segmentation but also provide strong technical support for agricultural intelligence.

#### 2.3.1. CNN-Based Methods

FCN [33] was the first to use a fully convolutional network instead of a fully connected network in a classification model. This approach solves the requirement that a fully connected network must use inputs of the same size and achieves the semantic segmentation of images of different sizes. However, it ignores global contextual information. To address this issue, ParseNet [39] introduces global information through global average pooling, which addresses the problem of FCN’s losing global information in semantic segmentation.

Although ParseNet takes global contextual information into account, it may lose some local details. To better capture both global contextual and detailed information, many scholars have made improvements based on the encoder-decoder structure. DeconvNet [40] introduces the deconvolution layer. Through the upsampling operation, it restores the high-resolution feature maps of the image, thus retaining more detailed information. SegNet [41] records the max-pooling indices during the encoder stage and uses these indices for the non-linear upsampling in the decoder stage. This effectively preserves the high-frequency details of the image while reducing the number of parameters and computational cost. HRNet [42] effectively preserves the detailed information of high-resolution features by fusing features of different resolutions from high to low and promotes information interaction among features of different resolutions. Therefore, many segmentation models adopt HRNet as the backbone network to enhance the model’s ability to capture contextual information. The design ideas of U-Net [34] and V-Net [43] in medical imaging, which deal with category imbalances through jump connections with loss function innovations such as Dice coefficients, have been migrated to complex scenarios of autonomous driving, such as enhancing the accuracy of the boundary segmentation between drivable areas and obstacles in rainy weather or nighttime illumination.

There have also been many achievements in the research of multi-scale information fusion and pyramid network models. FPN [44] was first used for object detection and later applied to semantic segmentation tasks. The FPN extracts high-level semantic information through a bottom-up path and then performs upsampling through a top–down path. It horizontally connects high-level features with low-level features to achieve feature fusion. PSPNet [45] proposed a PPM module that integrates four pyramid features of different sizes to aggregate contextual information from different regions.

The pyramid structure enables the model to have a sufficient receptive field, yet it struggles to capture spatial dependencies. The attention mechanism was first applied in machine translation. By enabling the model to automatically focus on the parts of the source sentence associated with the object word for prediction, it can effectively capture long-range dependencies. In tasks such as detection, diagnosis, and segmentation, the introduction of multi-scale features is often required. A highly effective approach is to integrate the attention mechanism into the fully convolutional network. This approach is commonly used not only in autonomous driving but also in other fields, such as the diagnosis of agricultural crop diseases and the detection of flowers and fruits [46,47,48], becoming a key technique to improve segmentation accuracy.

In terms of specific application patterns of attention mechanisms in semantic segmentation, they can be further divided into the following categories.

The spatial attention mechanism strengthens the focus on local regions by modeling the spatial dependency relationships among pixels. For instance, in PSANet [49], a bidirectional information propagation path is designed. The Collect branch aggregates global features to optimize its own predictions, while the Distribute branch spreads local features to assist the global predictions. Eventually, the global context and local features are fused through a residual connection. This approach breaks through the local perception limitations of traditional convolutional networks, enhances the modeling of long-range dependencies while preserving detailed information, and significantly improves the segmentation accuracy of small objects and boundaries in complex scenarios. The spatial attention mechanism is applicable to the segmentation of lane lines with complex pavement markings.

The channel attention mechanism focuses on screening the importance of feature channels and strengthens the key semantic channels. Taking PANet [50] as an example, it uses global average pooling to generate channel statistics and then maps them through a two-layer perceptron to obtain channel attention weights. Higher weights are assigned to the important channels. It is, therefore, suitable for tasks that require highlighting the importance of feature channels. This mechanism effectively improves the model’s utilization efficiency of semantic features and reduces the interference of redundant channels. The channel attention mechanism can be used to filter key semantic channels and reduce redundant feature interference when sharing a backbone network with an object detection task.

The hybrid attention mechanism combines the attention in the spatial and channel dimensions to achieve more comprehensive feature modeling. For example, DANet [51] deploys both a spatial attention module and a channel attention module on top of the FCN. The former locates the key spatial regions in the image, and the latter screens the important feature channels. Finally, the feature representation is further optimized by fusing the outputs of the two modules, enabling more precise semantic segmentation in complex scenarios. The hybrid attention mechanism can balance the feature demands of different tasks in a multi-task model.

The introduction of the attention mechanism inevitably increases the complexity of the model. To achieve a balance between detection accuracy and inference speed in the drivable area segmentation task, some lightweight drivable area segmentation networks have been proposed, such as ShuffleNet [52] and ENet [53]. ShuffleNet uses pointwise group convolution and channel shuffling to reduce computational cost, and ENet adapts to mobile devices through early downsampling and asymmetric convolution, both of which are suitable for real-time scenarios such as autonomous driving.

It is worth noting that the lightweight networks mentioned above, as well as many other models, employ the technique of inflationary convolution. This type of convolution effectively expands the receptive field by inserting voids within the convolution kernel without adding additional parameters or computational burden. These lightweight schemes often rely on multi-scale context aggregation (MSCA) [54] techniques to capture rich context information, and their core methods include dilated convolution, ASPP modules, and global average pooling layers. The DeepLab family of models significantly improves the performance of semantic segmentation by gradually introducing MSCA techniques. DeepLabv1 [55] fuses the techniques of DCNNs and probabilistic graphical models to integrate the output of the final layer of the DCNN with a fully-connected Conditional Random Field (CRF) [56] in order to address the problem of the insufficient localization properties of the DCNN’s final layer output in precise object segmentation. DeepLabv2 [57] introduces the ASPP module based on the MSCA to fuse features with multiple dilation rates. DeepLabv3 [58] removes the CRF post-processing by using a global average pooling layer. DeepLabv3+ [35] further combines the encoder-decoder structure to preserve details and gradually constructs an end-to-end framework for MSCA. Its efficient utilization of multi-scale features provides key technical support for the real-time collaboration of semantic segmentation, object detection, and lane detection in multi-task perception.

#### 2.3.2. Transformer-Based Methods

The global modeling ability of Transformer compensates for the local perception limitations of the CNN and demonstrates its advantages in complex traffic scenarios such as multi-lane situations and occlusions.

In Transformer-based semantic segmentation methods, the attention mechanism also serves as a core pillar, supporting the model’s deep modeling of global dependencies.

After dividing an image into multiple patches, ViT [59] captures the correlations between different patches via the self-attention mechanism, introducing a new approach to image classification tasks. Its success demonstrates the potential of the attention mechanism in global modeling. SETR [60], using ViT as a backbone, is the first visual Transformer-based semantic segmentation model. It replaces the traditional CNN encoder with an encoder of a pure Transformer structure, thus changing the architecture of the semantic segmentation model. However, ViT can only output low-resolution features at a single scale, so PVT [61] proposed a pyramidal Transformer that can be applied to dense prediction. It generates multi-scale features through hierarchical self-attention, which not only retains the advantage of CNNs in processing multi-scale information but also strengthens the global dependency modelling with the help of Transformer’s self-attention, so it can be used as a backbone for different tasks. The hierarchical Transformer architecture of SegFormer [62] employs self-attention without positional encoding, circumventing the interpolation loss issue inherent in traditional Transformers. Its encoder efficiently fuses multi-scale features through self-attention, while the decoder aggregates information using a lightweight MLP, achieving an excellent balance between accuracy and efficiency in scenarios such as autonomous driving. HRFormer [63] leverages the multi-resolution parallel design of HRNet and combines it with local window self-attention. While enhancing memory and computational efficiency, it learns high-resolution representations through the attention mechanism, precisely adapting to dense prediction tasks. These models, centered around the attention mechanism, fully demonstrate the powerful ability of Transformer in capturing long-range dependencies and integrating global information. They complement the applications of attention mechanisms based on CNNs, further improving the comprehensive coverage of attention mechanisms in common semantic segmentation algorithms.

However, it is worth noting that not all Transformer-based models rely exclusively on traditional attention mechanisms. PoolFormer [64] uses a simple spatial pooling module instead of the attention module to implement information interaction between tokens, which also turns out to work well. This proves that the success of Transformer does not only depend on specific self-attention or MLP modules but more on its generic architecture. This generic architecture is MetaFormer [64], which allows different models to be generated by specifying different token mixers.

#### 2.3.3. Hybrid Architectures

There are also some studies that combine Transformers with CNNs to form hybrid architectures. TransUnet [65] combines Transformer with U-Net. It takes the tokenized image patches of the CNN feature maps as the input sequence, utilizes the Transformer to extract global features, and then combines the encoded features with high-resolution CNN features through a cascaded upsampler to achieve precise localization. MixFormer [66] uses a convolutional layer in the backbone to extract local features, after which the features are globally modelled by Transformer’s self-attention, and the two are combined to improve the segmentation performance in complex scenes. This cross-architecture application of attention gives full play to the strengths of different modules and drives semantic segmentation towards better performance.

#### 2.3.4. Novel Architectures

Although CNNs and Transformers dominate the field of computer vision, researchers are still attempting to build network architectures using only MLPs to further explore the potential of visual networks. Tolstikhin et al. [67] proposed MLP-Mixer, a mixer structure fully based on MLPs. Chen et al. [68] further explored the application of MLPs in dense prediction tasks and proposed the CycleMLP structure, which enhances feature interactivity through cyclically permuted MLP blocks. In order to better capture the correlations of local features, Lian et al. [69] introduced an axial shift strategy and designed AS-MLP, providing a new perspective for MLP-based visual networks. Some novel MLP-based architectures have also attempted to incorporate variants of the attention mechanism. For example, they improve the MLP’s ability to model spatial semantics through positional attention or channel attention. Although such explorations are relatively new, they have demonstrated the potential to optimize the modeling of feature relationships.

### 2.4. Lane Detection

Lane detection is an important task in the environmental perception of autonomous vehicles. Similarly, in the field of agricultural robots, perception technologies for object detection and navigation line detection are crucial as well. For example, the SN-CNN model for agricultural robots [70] achieves high-precision navigation line detection and obstacle recognition by optimizing the network architecture and introducing the attention mechanism. Ma et al. [71] proposed a method for extracting navigation lines and measuring pedestrian obstacles through YOLOD-SLAM2, which improves the YOLOv5 algorithm, and a distance measurement algorithm based on a four-region depth comparison, realizing the detection of tree trunks between left and right rows in orchards.

Currently, there are mainly two feature extraction methods for vision-based lane detection tasks: one is the traditional lane-line feature extraction method, and the other is the deep learning-based method.

Traditional lane detection methods rely on highly specialized manual designs. They utilize features such as the color, gradient, texture, and vanishing point of the lane lines to separate the lane lines from the images. Firstly, they perform preprocessing operations like smoothing and denoising on the images to extract the regions of interest. Subsequently, they use the manually designed features to extract the edge information of the lane lines from the preprocessed images. Finally, the specific information of the lane lines is determined by fitting these extracted features.

The mainstream algorithms for traditional lane detection mainly include those based on the Hough transform, those based on the LSD straight line, those based on the top view transformation, those based on fitting, and those based on the parallel perspective vanishing point.

In complex road conditions, such as lane marking wear, illumination changes, and rainy or foggy weather, traditional handcrafted feature methods struggle to achieve effective detection. In contrast, deep learning approaches, owing to their stronger feature capture and generalization capabilities, have been widely adopted in lane detection tasks.

There are mainly five types of lane detection methods based on deep learning: the method based on segmentation, the method based on anchors, the method based on row classification, the method based on key points, and the method based on parametric curves.

#### 2.4.1. Segmentation-Based Method

The segmentation-based method transforms lane detection into a pixel-by-pixel classification problem. It uses models such as CNN or Transformer to directly extract the lane line features from the image and to perform pixel-level classification, dividing each pixel into either the lane line area or the background. Consequently, the classic algorithm SCNN [72] treats the rows or columns of the feature map as layers. Through convolution calculations, non-linear activations, and summation operations, it enables information to propagate among neurons in the same layer, constructing a spatially deep neural network. However, due to its large backbone network, SCNN has a slow speed of only 7.5 FPS, which limits its applicability in practical applications. In response to the problem of slow data transfer in SCNN and the possible loss of information during long-distance propagation, RESA [73] introduced an innovative slicing strategy. It slices the feature map in both vertical and horizontal directions and uses different set strides for cyclic shift operations, effectively aggregating the data from different sliced feature maps. In this way, it can not only transmit spatial information more directly and efficiently across the entire feature map but can also enhance the model’s ability to capture details, ensuring the integrity and accuracy of the information transfer. The segmentation-based method, as a basic scheme for pixel-level classification, provides fine pixel-level judgments for lane detection, and its feature extraction method can share the underlying features with tasks such as drivable area segmentation, which is an important foundation for achieving a semantic understanding of the scene in multi-task perception.

#### 2.4.2. Anchor-Based Method

The anchor-based semantic segmentation method presets a series of anchor points in the image for locating and recognizing target objects. Then, it uses a regression algorithm to adjust the candidate boxes generated by these anchor points to more accurately match the target objects. LaneATT [74] designed anchors with an elongated shape and proposed an anchor-based global information aggregation attention mechanism. The backbone network of this model uses ResNet to generate feature maps and extract features, thus gathering the features of each anchor point. These features are then combined with the features generated by a set of global attention modules and jointly passed to two fully connected layers to predict the angles and coordinates and finally output the lane lines. By integrating local and global features, LaneATT can effectively utilize the information from other lanes, which is particularly crucial in multi-task scenarios where there is occlusion or a lack of obvious lane markings. UFLD [75] introduced the row classification technique for the first time. By adopting the row anchor method, it achieved an ultra-fast lane detection speed. At the same time, it introduced a structure-aware module to improve the detection accuracy. The efficient detection speed of the anchor-based method is suitable for parallel processing with other tasks and meets the real-time requirements of embedded multi-task systems.

#### 2.4.3. Row Classification-Based Method

The row classification-based semantic segmentation method is a strategy that decomposes the image segmentation task into a line-by-line processing approach. By analyzing the content of the image line by line, it achieves the classification of pixels in each row, thus completing the semantic segmentation task. CondLaneNet [76] is a new type of lane detection framework that focuses on instance-level lane detection. Through conditional convolution and a line-by-line formulation strategy, it dynamically predicts the shape of each lane line instance. For complex topological structures, such as dense lane lines and bifurcated lane lines, CondLaneNet introduces the Recursive Instance Module, which effectively solves these problems. This method adopts an end-to-end pipeline design, requiring almost no post-processing, and has high real-time performance. Excellent at handling complex scenes, this end-to-end design facilitates integration with other tasks and improves multi-task fluency.

#### 2.4.4. Key Point-Based Method

Traditional methods mostly adopt a top-down strategy, using predefined anchor points to regress lane lines of different shapes. However, due to the fixed shapes of the anchor points, this method has difficulty adapting to the complex and changeable shapes of lane lines. Inspired by human pose estimation, researchers have begun to transform lane detection into a key point estimation problem to more flexibly capture the shape features of lane lines. GANet [77] adopts a more effective post-processing method, which neither requires feature embeddings nor local associations for clustering or reconstructing the entire lane. Each key point finds its corresponding lane by adding its offset coordinates to the starting point of the lane line in a parallel manner, instead of expanding point by point. This is the first method to regress key points in a global way, which is more effective than local regression, and its efficient post-processing reduces the computational burden and improves the overall efficiency in multi-task perception.

#### 2.4.5. Parametric Curve-Based Method

For autonomous driving applications, lane detection algorithms ultimately need to output the geometric model of lane lines for the subsequent decision-making and planning tasks. The traditional method requires model fitting after extracting the lane line pixels, whereas the parametric curve-based method outputs the model directly. The classic network is PolyLaneNet, which takes the image from the front-view camera installed in the vehicle as the input and outputs the polynomials representing each lane marking in the image, as well as the domain of these polynomials and the confidence score of each lane. “Rethinking Efficient Lane Detection via Curve Modeling” [78] proposed a method for lane detection in RGB images based on parametric Bezier curves. This method simulates the geometric shape of lane lines by using parametric Bezier curves, solving the optimization difficulties of existing complex and changeable polynomial curve methods. In addition, this study proposed a feature flipping fusion module based on deformable convolutions. The symmetry of the driving scene is used to enhance the feature representation and improve the detection accuracy, and this direct output of the geometric model can be used in a multi-task framework to provide direct support for tasks such as path planning, reducing the cost of conversion between tasks.

## 3. Multi-Task Perception

### 3.1. Multi-Task Learning

In industry, there is often a desire for a single machine to be able to perform multiple tasks. For example, a multi-task robot transplanting workstation for greenhouse seedlings [79] can automatically pick up and plant a whole row of seedlings through a transplanting system with grippers. It moves the seedling trays and flowerpots to designated positions via two conveyor belts and uses a filling unit to automatically fill the soil and punch holes. Additionally, it has a control system that coordinates the work of each part through a PLC, enabling it to complete the transplanting task efficiently and accurately, which has a high practical value.

This is the case for mechanical design and automatic control, and it is also true for software network learning aspects such as image recognition and natural language processing. However, in most existing studies, data learning strategies based on deep learning often tend to be similar, and most of them adopt the method of single-task learning. Although this approach, which processes different categories of tasks through different deep learning networks, can meet the self-learning and modeling needs of most data, it requires training multiple independent models to predict multiple attributes. This not only leads to cumbersome operations such as the frequent adjustment of the model structures and parameter tuning among different tasks but also brings inconvenience to practical applications. Therefore, if the same deep learning network can be used to achieve the learning and modeling of multiple types of tasks, the efficiency and accuracy will be significantly improved. Against this background, multi-task prediction has become particularly necessary in practical industrial environments, thus promoting the rise of multi-task learning strategies [80].

Multi-task learning is a classic inductive transfer mechanism in the field of machine learning. Its core objective is to utilize the useful information implicit in multiple related tasks to enhance the generalization ability of the model. Specifically, multi-task learning conducts parallel training through a sharing mechanism [81]. This mechanism enables the model, while learning one task, to leverage the shared information to learn and acquire knowledge of other related tasks. In this way, it strengthens the model’s comprehensive understanding and processing capabilities for different tasks.

**Figure 3:**
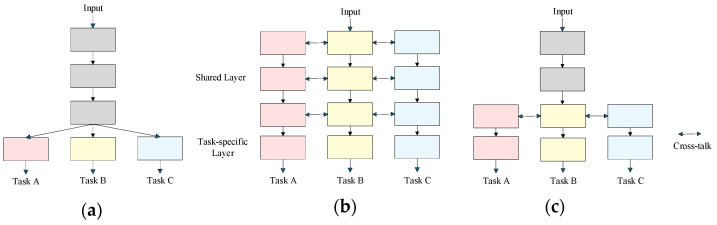
Classification of multi-task learning sharing mechanisms: (**a**) hard parameter sharing; (**b**) soft parameter sharing; and (**c**) hybrid sharing.

Common sharing mechanisms in multi-task learning include hard parameter sharing, soft parameter sharing, and hybrid sharing, as shown in Figure 3.

Hard parameter sharing is the most commonly used multi-task learning method in deep learning, whose core lies in sharing the underlying hidden layer parameters across all the tasks while retaining several task-specific output layers. Specifically, multiple tasks share the same set of base network parameters, mining shared features across tasks by maximizing parameter sharing. For example, in image multi-task learning, object detection and semantic segmentation often share the bottom convolutional layers of a CNN to extract universal features like edges and textures. Specifically, independent task-specific branches are designed in the output layers. The detection task employs a bounding box regression head to output object locations, while the segmentation task uses a pixel classification head to generate per-pixel predictions. This approach significantly reduces the total number of parameters and the computational overhead, accelerating the training. However, when tasks differ significantly, mutual interference may occur due to feature competition, such as conflicts in shared layers between the semantic abstraction required for detection and the spatial details required for segmentation. YOLOP [82] represents a typical implementation of the hard parameter sharing paradigm.

Soft parameter sharing means that each task has its own model, and each model has its own independent parameters. The differences between these independent parameters are regularized, thus encouraging them to maintain a certain degree of similarity. This regularization mechanism helps to achieve collaborative learning across tasks while preserving the task-specific features. For example, in multi-task scenarios of natural language processing, distinct language tasks such as text classification and named entity recognition maintain independent network parameters, yet regularization is employed to enforce similar parameter distributions. This enables the model to learn task-specific features while leveraging information from other tasks. In contrast to hard parameter sharing, soft parameter sharing offers greater flexibility when tasks differ significantly, avoiding the conflicts caused by strict parameter sharing. However, this approach increases the total number of parameters and computational costs.

Hybrid sharing strategies integrate the advantages of hard and soft parameter sharing through a hierarchical parameter design. The early network layers share weights to learn universal task features such as low-level visual features like edges and textures in visual tasks, realizing hard parameter sharing. The later layers are independently designed for each task to capture complex task-specific features such as object semantic features for detection tasks and spatial detail features for segmentation tasks, forming an effect similar to soft parameter sharing. This layered design not only utilizes task commonalities to improve efficiency but also reserves flexible space for task specificity. For example, in HybridNets [83], the bottom layers adopt a lightweight EfficientNet [84] backbone with shared weights to extract universal low-level features such as the sedges and textures from images, achieving hard parameter sharing. The subsequent detection and segmentation heads are independently designed, with the detection head processing object semantic features and the segmentation head focusing on capturing spatial detail features, creating an effect analogous to soft parameter sharing.

The core distinction between hybrid sharing strategies and hard parameter sharing lies in their approaches to parameter utilization. Hybrid sharing involves hierarchical and selective parameter sharing, incorporating both shared components to extract universal features and independent or differentiated designs to adapt to task characteristics. In contrast, hard parameter sharing fully shares bottom-layer parameters across all the tasks, with only the task-specific output layers remaining independent.

When selecting a parameter sharing strategy for multi-task learning, factors such as task correlation and task types need to be comprehensively considered. When tasks are highly correlated, hard parameter sharing becomes an ideal choice due to its efficient parameter utilization, fully leveraging the commonalities between tasks. When task correlation is moderate, hybrid sharing strategies can balance efficiency and flexibility while reducing task conflicts. When task correlation is low, soft parameter sharing or dynamic parameter generation is more advantageous, better adapting to task differences. From the perspective of task types, the CV field often uses hybrid sharing strategies to meet the diverse needs of feature extraction and processing in visual tasks, while the NLP field more frequently employs soft parameter sharing to capture complex semantic correlations and differences between text tasks, achieving more precise task processing.

Therefore, for two targets with a certain degree of correlation, the prediction performance of a multi-task CNN is better than that of a single-task CNN. We will further explore how these sharing mechanisms work in practical applications through some specific multi-task learning methods and model examples.

### 3.2. Environment Perception Technology Based on Multi-Task Network

Mask R-CNN [85] adds an additional branch for predicting object masks on the basis of Faster R-CNN, enabling instance segmentation while performing object detection. The input image passes through the ResNet, a CNN network, to obtain feature maps. The RPN operation is performed on the feature maps to obtain the Region of Interest. RoI Align is used to filter and fine-tune the RoI and input it into two branches. Two fully connected layer branches are used for classification and regression, and the convolutional layer is used for segmentation.

Cascade R-CNN [86] is an improvement based on Mask R-CNN, using a cascade operation to handle the multi-scale information of object instances.

Mask Scoring R-CNN [87] adds a new head on the basis of Mask R-CNN, which is used to learn and predict the quality of the instance segmentation results. This quality is measured by the IoU between the predicted mask and the ground truth mask. Specifically, Mask Scoring R-CNN multiplies the predicted mask IoU by the classification score to evaluate the mask score.

OmniDet [88] is a multi-task visual perception system for surround-view fisheye lenses. It integrates six major tasks, including depth estimation, visual odometry, semantic segmentation, motion segmentation, object detection, and lens contamination detection.

MultiTask-CenterNet [89] enhances the anchor-free method of CenterNet and is used to train multiple different perception-related tasks together, including object detection, semantic segmentation, and human pose estimation. Compared with the single-task network, this multi-task network reduces the inference time and network size.

YOLO-Pose [90] completes 2D multi-person pose estimation based on the YOLOv5 framework structure. The backbone network uses CSPDarknet, and the neck network uses PANet [47]. The detection heads of four scales all output bounding boxes and key points.

LSNet [91] summarizes object detection, instance segmentation, and pose estimation as position-sensitive visual recognition and uses a unified solution to handle these tasks.

The Transformer model is a general-purpose visual feature extractor and can be extended into a multi-task network for handling multiple tasks simultaneously. Research teams have gradually improved the performance of the model by optimizing the attention mechanism. In 2021, Wang et al. [61] proposed a multi-task network PVT v1 that is fully based on Transformer. It innovatively integrates an asymptotically shrinking pyramid structure with a spatial reduction attention module, effectively reducing the computational resource requirements of ViT [59] in high-resolution feature learning. In the same year, Liu et al. [92] proposed Swin Transformer v1. Through the shifted window attention mechanism, it confines the self-attention operation to local non-overlapping windows while retaining the ability to interact information across windows.

In subsequent improvements, in 2022, PVT v2 [93], designed by Wang et al., integrates a linear complexity attention layer, overlapping patch embedding, and a convolutional feed-forward network, optimizing the computational complexity to a linear level. It demonstrates significant performance advantages in four fundamental visual tasks: object classification, object detection, instance segmentation, and semantic segmentation. However, this architecture is still limited by the feature extraction mode of a single encoder, which affects the sufficiency of the feature extraction effect. In the same year, the SepViT [94] architecture developed by Li et al. innovatively integrated a depthwise separable self-attention mechanism. Through the collaborative design of the window “token” embedding technology and the grouped attention strategy, it constructs a cross-window attention correlation model while maintaining a low computational cost. This solution not only enables the efficient interaction of features within local windows but also establishes a global information conduction path across windows, significantly enhancing the multi-scale feature modeling ability. The Swin Transformer v2 [95] improved by Liu et al. in the same period solves the problems of the unstable training of Swin Transformer v1, the resolution difference between pretraining and fine-tuning, and the dependence on labeled data. It has become the largest dense visual model to date.

Aiming at the characteristic that segmentation tasks require the refinement of target edge information, existing methods still face challenges in edge refinement, and the problem of interference from non-edge features within the window urgently needs to be solved. In order to improve the performance of the Transformer when applied to multi-task networks, people have begun to devote themselves to exploring more effective attention mechanisms. In 2021, Chu et al. [96] proposed the spatial separable self-attention mechanism and the cross-window self-attention mechanism. Based on these, they proposed the Twins-PCPVT and Twins-SVT multi-task backbone networks and achieved superior performance. In 2022, Dong et al. [97] proposed a multi-task backbone network CSWin Transformer with a window attention mechanism and introduced local enhanced position encoding technology. This optimized the multi-task processing ability while reducing the computational cost. However, the problem of information attenuation in cross-stage feature transfer and the phenomenon of performance degradation when transferring the attention mechanism still restricts the further improvement of the model’s performance.

## 4. Multi-Task Perception for Autonomous Driving

### 4.1. Classic Network Framework

Early multi-task networks focused on shared encoder designs, enabling cross-task feature reuse through task-specific decoders, yet they exhibited notable differences in task conflict resolution and efficiency optimization. As the first encoder–decoder multi-task framework, MultiNet [98] employed VGG16 and ResNet as shared encoders to extract universal image features, providing a foundational representation for subsequent tasks. For object detection, it used a RoI Align layer to extract regional features and designed a dual-branch classification decoder to adapt to different input resolutions, enabling target category judgment. For semantic segmentation, it adopted the FCN [33] structure as a task-specific decoder, performing operations such as upsampling on features from the shared encoder to achieve the pixel-level segmentation of road regions. Its core contribution validated the feasibility of multi-task shared backbones. However, its disadvantages were also quite prominent. The independent decoders it employed limited the co-optimization of tasks, and it had a fixed input size, which reduced its adaptability to various scenarios. Additionally, it lacked a multi-scale detection capability, making it difficult for it to handle complex scenes with diverse scales, and suffered from segmentation computing redundancy, increasing an unnecessary computational overhead.

Addressing the feature fusion limitations of MultiNet, DLT-Net [99] introduced a feature pyramid and context tensor mechanism. It fused shallow and deep features from VGG16 [100] via element-wise mean operations, preserving shallow geometric details while enhancing deep semantic representations. Each task branch was equipped with a specific decoder for the subsequent processing. The traffic object detection branch used a focal loss function in its decoder to effectively address class imbalance and improve small-object detection accuracy. The drivable area segmentation and lane detection branches leveraged the fused feature maps in their decoders to achieve precise spatial segmentation and lane detection. A notable advantage was its context tensor for cross-task feature fusion, which facilitated a better information exchange between tasks, along with high multi-task precision balance and strong robustness in sparse scenarios, making it more reliable in less dense environments. Nevertheless, it had some drawbacks. Its performance on Jetson TX2 was limited (<15 FPS), making it less suitable for such platforms. The model was complex and had a high training resource consumption, which posed challenges for practical applications. Moreover, it showed poor generalization on long-tailed data, reducing its effectiveness in certain scenarios.

The breakthrough in real-time multi-task frameworks began with YOLOP [82]. In the encoder part, the backbone network was based on YOLOv4 [16], using CSPDarknet as a shared encoder to extract multi-scale image features. These features were further processed by the neck network composed of the SPP [11] module and the FPN [44] module, enabling the fusion of features at different scales and semantic levels. The detection head, serving as a task-specific decoder, was connected to the FPN in the neck network, which transmitted semantic information from top to bottom. Then, it utilized the bottom-up PAN [50] to transmit the localization features. After fusion, object detection was carried out based on the multi-scale fused feature maps of PAN, outputting class probabilities and prediction confidences. For the decoders of drivable area segmentation and lane segmentation, after the low-level features of the FPN were input, they passed through three upsampling layers using nearest neighbor interpolation to achieve a high-precision segmentation output. This architecture achieved an inference speed of 41 FPS on the BDD100K dataset. It brought several advantages, such as real-time triple-task processing on Jetson TX2 (23 FPS), which was suitable for practical applications; a lightweight design for edge deployment, reducing resource requirements; and an adaptive cascade module that optimized the segmentation results. However, the sharing of deep features led to the loss of geometric details. The high-resolution features required for segmentation tasks were diluted during the downsampling process of the backbone, resulting in a decrease in the lane line positioning accuracy under complex road conditions. Additionally, there were other limitations. The fixed feature allocation strategy introduced a multi-task module delay, affecting real-time performance. It also caused accuracy fluctuations in complex scenes and made it necessary to trade off between accuracy and real time, limiting its overall effectiveness in some cases.

Subsequent lightweight designs, such as A-YOLOM [101] and HybridNets [83], focus on efficiency optimization.

A-YOLOM [101] builds a lightweight architecture based on the YOLOv8 backbone network. Its core innovation lies in the differential design of the detection neck and the segmentation neck. The detection branch uses the PAN [50] of YOLOv8. Through bottom-up feature fusion, it strengthens the target positioning information to meet the high-precision requirements of the detection task for bounding box coordinates. The segmentation branch adopts the FPN [44] and introduces an adaptive cascade module. This module dynamically fuses features from different stages of the backbone network through learnable weights. This design offered several advantages, including an ultra-lightweight architecture (<3 MB), which was very suitable for resource-constrained environments; adaptive multi-task weight allocation to better balance different tasks; and support for a dynamic resolution input, increasing its flexibility. This design enables A-YOLOM to maintain real-time performance on the BDD100K dataset while improving the boundary positioning accuracy of lane segmentation by 3.2% compared with YOLOP [82]. However, it still had some shortcomings, similar to others. Its performance on Jetson TX2 was limited (<15 FPS), and it faced issues like complex models, high training resource consumption, and poor generalization on long-tailed data, which restricted its application scope.

HybridNets [83] adopts an architecture with a shared encoder and independent decoders. It combines lane segmentation and drivable area segmentation into a unified road element segmentation task. By using the lightweight EfficientNet-B3 [84] as the shared encoder, it reduces the model complexity. The neck network introduces the BiFPN [102] of EfficientDet, replacing the traditional unidirectional FPN with cross-scale bidirectional connections to achieve efficient feature fusion and weight optimization. The detection head, as a task-specific decoder, receives the multi-scale fused features output by BiFPN to perform the object detection task. The segmentation head, as another task-specific decoder, takes the high-resolution P2 layer features from the shallow part of the backbone network, reducing redundant calculations and improving the efficiency of feature reuse. In terms of the loss function design, the detection branch uses focal loss to handle class imbalance and foreground detection and cooperates with the optimized smooth L1 loss for bounding box regression. The segmentation branch combines the focal loss and the Tversky loss to balance pixel-level classification accuracy and class imbalance. It had distinct advantages, such as a hybrid CNN-Transformer architecture that combined the strengths of different models, multi-modal fusion for richer feature representation, and end-to-end multi-task optimization to improve overall performance. However, it also had notable drawbacks. It had high computational requirements (>16 GB VRAM), making it difficult to deploy on edge devices with limited resources, and slow training convergence, which prolonged the training process. Moreover, task merging caused optimization objective conflicts; for example, the unified segmentation head had difficulty balancing the fine-grained boundary detection of lane lines and the large-area semantic classification of drivable areas, leading to limited generalization ability in complex marking scenarios.

Both methods promote the lightweight design of multi-task models through architectural innovation. The differential neck design of A-YOLOM performs better in balancing accuracy and speed, while the task-merging strategy of HybridNets is more efficient in specific scenarios such as simple road structures. Together, they reveal the inherent trade-off between feature specificity and computational efficiency in multi-task framework design. The former improves accuracy through structural segmentation, and the latter reduces complexity through task integration. However, they both need to find the optimal solution between task-specific requirements and the generality of shared features.

YOLOPv2 [103] achieves performance breakthroughs based on multi-task frameworks such as YOLOP [82] and HybridNets [83] through feature hierarchy decoupling and dynamic loss balancing, achieving a state-of-the-art balance between real-time performance and accuracy on the BDD100K [104] dataset. YOLOPv2 introduces core improvements through structural and functional innovations. The backbone network employs E-ELAN [105] constructed with grouped convolutions, dividing feature channels into two groups via cross-stage connections. This design reduces the parameters by 32% while enhancing feature diversity. The neck network retains the SPP [11] and FPN [44] structures, generating multi-scale features through spatial pyramid pooling and implementing differentiated feature allocation according to task characteristics. The drivable area segmentation branch connects to shallow P2-layer features of the FPN to preserve high-resolution geometric information, while the lane segmentation branch receives deep P5-layer features to strengthen semantic perception, addressing the geometric distortion issue in YOLOP caused by shared deep features. In the multi-task head design, the detection head uses the PAN [50] structure to fuse bottom-up positioning features and top-down semantic features. It incorporates scene-adaptive anchor boxes generated via K-means clustering and optimizes detection performance through a combined loss function. The drivable area segmentation adopts a U-Net [34] architecture, and the lane segmentation introduces deconvolution layers and channel attention modules, addressing class imbalance with cross-entropy loss and Dice-Focal hybrid loss, respectively. Regarding training strategies, data diversity is enhanced via Mosaic and Mixup augmentation, combined with a four-stage training pipeline (COCO [106] pretraining, multi-task adaptation, hard example mining, and quantization compression). The final model reduces the volume by 75% compared to its predecessor, increases the inference speed by 85.7%, and achieves a detection mAP50 of 83% and a segmentation mIoU of 93%, providing an efficient solution for vision-based autonomous driving perception.

After a detailed analysis of each model above, we can summarize their advantages and disadvantages, as shown in Table 1 below.

The performance effects achieved by various multi-task models in different tasks such as traffic object detection, drivable area segmentation, and lane detection are not the same. Their specific experimental results on the BDD100K dataset are presented in Table 2, Table 3 and Table 4. Regarding the detailed experimental settings of each model, including the input image size, backbone architecture, and neck network configuration, interested readers are advised to refer to the corresponding references. For more specific experimental information, such as the training parameters and data processing procedures, it is recommended to consult the original research publications cited throughout the review and source code for in-depth exploration.

### 4.2. Loss Function

The loss function is the core part of training a deep learning model, and it directly affects the learning effect and final performance of the model. In multi-task learning, since it involves the joint training of multiple related tasks, the design of the loss function is particularly important. Multi-task learning jointly trains multiple related tasks, and its total loss function is a weighted summation of the loss functions of each task, as shown in Formula (1).

(1) $$L=\sum_{i=1}^{n}\omega_{i}L_{i}$$

where $L_{i}$ represents the loss of the *i*-th task, and $\omega_{i}$ is the corresponding weight coefficient for the loss of the *i*-th task.

In the multi-task perception of autonomous driving, the common tasks are traffic object detection, drivable area segmentation, and lane detection. The loss function is composed of these three parts, and its calculation is as follows:

(2) $$L=\omega_{1}L_{det}+\omega_{2}L_{segda}+\omega_{3}L_{segll}$$

where $L_{det},L_{segda}$, and $L_{segll}$ are the losses for traffic object detection, drivable area detection, and lane line detection, respectively. Meanwhile, $\omega_{1},\omega_{2},\omega_{3}$ are the corresponding weight coefficients for the losses of these tasks.

The importance of different tasks is not entirely the same. Therefore, it is necessary to assign a weight to each task. The assignment of weights can be based on factors such as the difficulty of the task, the amount of data, and the correlation between tasks. There are mainly three methods for setting the weights of the loss function: manually setting the weights through experience or experiments, dynamically adjusting the weights according to the training progress of the tasks, and automatically learning the weights using an auxiliary network.

Each task has its specific loss function. Selecting an appropriate loss function is crucial for the performance of each task. The loss function of traffic object detection is generally divided into three parts: classification loss, bounding box loss, and IoU loss. For the classification loss, the focal loss or cross-entropy loss is commonly used, and for the bounding box loss, the L1 loss is often applied. The loss functions for drivable area and lane segmentation are divided into classification loss and IoU loss. The focal loss or cross-entropy loss is commonly used for the classification loss. The focal loss ensures that the model will not become overwhelmingly biased towards the dominant and easily learnable classes, while the cross-entropy loss minimizes the classification error between the pixels of the network output and the targets.

### 4.3. Emerging Architecture

While classical frameworks have laid the foundation for multi-task perception, breakthroughs in two technical paradigms in recent years are reshaping the field. Novel neural models such as Transformer and its improved architecture Mamba break through performance bottlenecks through global dependency modeling and efficiency optimization. Meanwhile, automated design methodologies, including Neural Architecture Search (NAS) and the AutoMTL framework, realize the algorithm-driven intellectualization of architectural design. These two paradigms complement each other, driving multi-task perception toward greater efficiency and robustness.

#### 4.3.1. Global Dependency Modeling with Transformers

In the field of multi-task visual perception, traditional methods primarily rely on CNNs as the backbone architecture. Although CNNs exhibit strong local feature extraction capabilities, the local connectivity of their convolutional operations inherently limits their ability to model global contextual relationships, leading to performance bottlenecks. To address this problem, researchers have explored integrating Transformer architectures into multi-task perception, leveraging the modeling advantages of self-attention mechanisms for long-range dependencies to break through the constraints of traditional frameworks.

CUTransNet [108] constructs an encoder-decoder architecture to achieve the deep fusion of CNN-derived local details and Transformer-based global semantics. It extracts multi-scale visual features to preserve low-level information such as edges and textures, while the Transformer encoding module processes low-resolution features in blocks, capturing global contextual dependencies through multi-head self-attention to compensate for CNNs’ limitations in modeling long-range relationships. The CUT module employs a U-shaped structure to fuse cross-resolution features, efficiently aggregating the global features generated by the Transformer with multi-scale features from the backbone network via upsampling, 3 × 3 convolutions, and skip connections to enhance spatial detail recovery. The detection head processes multi-scale features using an anchor-based mechanism, generating adaptive anchors via K-means clustering to enable the precise detection of small objects. The semantic segmentation head merges the lane and drivable area segmentation tasks, using a feature pyramid network to produce high-resolution predictions. On the BDD100K dataset, this architecture achieves real-time inference at 50 FPS, with a small-object detection mAP50 of 84.6% and drivable area segmentation mIoU of 91.6%, validating the effectiveness of heterogeneous feature fusion.

In vision-based 3D perception scenarios, BEVFormer [109] constructs bird’s-eye view (BEV) feature representations through a spatiotemporal Transformer architecture, addressing the challenge of spatiotemporal information fusion across multiple cameras. Its spatial cross-attention module uses learnable grid-shaped BEV queries to project 3D spatial reference points onto 2D camera views, focusing on RoIs via deformable attention to efficiently aggregate multi-camera spatial features while reducing computational costs. The temporal self-attention module aligns historical BEV features using ego-motion, recursively fusing temporal information to enhance a dynamic object velocity estimation and occluded object detection, avoiding the temporal information redundancy of traditional stacking methods. This framework achieves an NDS of 56.9% on the nuScenes dataset, a 9.0% improvement over previous state-of-the-art methods. Notably, it significantly enhances object recall and localization accuracy in low-visibility scenarios, providing an efficient paradigm for end-to-end 3D detection and map segmentation. These advancements demonstrate that Transformers, by complementing CNNs, are driving breakthroughs in accuracy, efficiency, and adaptability for multi-task perception in complex scenes, serving as a critical technical pathway connecting local feature extraction and global semantic understanding.

#### 4.3.2. Sequence Perception and Multi-Task Processing with Mamba

Addressing the quadratic complexity bottleneck of traditional Transformers in long-sequence processing, the Mamba framework based on state-space models (SSMs) achieves technical breakthroughs through linear time complexity and a structured design. Mamba initializes the state transition matrix using the HiPPO algorithm, constructing a structured state space to capture long-term sequence dependencies. This enables the efficient processing of million-length sequences, avoiding the computational explosion of self-attention mechanisms. It introduces a dynamic input gating mechanism to reduce computational costs by adaptively filtering redundant information while enhancing feature modeling accuracy. Optimized for modern GPU hardware, it achieves high hardware utilization through parallel associative scanning and memory reconstruction techniques, improving inference speed by two-five times compared to Transformers of the same scale. These characteristics make it highly advantageous for processing long-sequence data such as radar point clouds and camera video frames in autonomous driving, capturing cross-frame dependencies through an efficient state-space mechanism to provide precise temporal modeling for dynamic scene perception. Especially in long-tail scenarios with complex weather or lighting changes, its strong generalization significantly reduces the performance degradation of multi-task perception models.

In the field of multi-task dense scene understanding, the MTMamba [110] architecture extends Mamba’s advantages to visual tasks, achieving the collaborative optimization of long-range dependency modeling and cross-task interaction through an encoder-decoder framework. Using a pretrained Swin Transformer as the encoder to extract multi-scale visual features, the decoder innovatively designs self-task Mamba (STM) blocks and cross-task Mamba (CTM) blocks. The STM block unfolds image features into one-dimensional sequences in four directions, leveraging Mamba’s state-space model to capture global spatial dependencies within individual tasks and selecting effective features via input-dependent gating to strengthen task-specific long-range semantic representations. The CTM block generates global shared representations by concatenating multi-task features, achieving the adaptive fusion of cross-task knowledge through task-specific features and dynamic gating mechanisms to avoid information redundancy and negative transfer. In the hierarchical design of the decoder, the patch expansion layer first restores feature resolution and fuses multi-scale information. After STM enhances the single-task context, CTM integrates cross-task features, ultimately improving the performance on datasets like PASCAL-Context by 2.08 mIoU in semantic segmentation and 5.01 mIoU in human parsing, validating the effectiveness of state-space models in visual multi-task scenarios.

For autonomous driving 3D detection tasks, MambaBEV [111] focuses on temporal fusion and global modeling in BEV perception. It achieves breakthroughs by utilizing the Temporal Mamba module and Mamba-based DETR detection head. Multi-scale features are first extracted from multi-camera inputs via the backbone network and FPN, with BEV feature maps generated through spatial cross-attention. The Temporal Mamba module aligns and compresses the historical and current BEV features, discretely rearranging them into one-dimensional sequences suitable for Mamba2 processing in four directions. By leveraging the linear complexity advantage of state-space models, it captures long-range spatiotemporal dependencies and enhances global contextual representations through skip connection fusion. The detection head processes the object query features with Mamba2, replacing part of the self-attention mechanism to improve multi-object interaction efficiency while refining spatial positioning with deformable attention. This design effectively addresses the insufficient global interaction of traditional deformable attention in large-object detection. It achieves an NDS of 51.7% and an mAP of 42.7% on the nuScenes dataset, significantly improving large-object detection accuracy and temporal consistency compared to similar methods. It also provides an efficient paradigm for real-time BEV perception.

In summary, Mamba and its derivative architectures demonstrate cross-domain adaptability in long-sequence modeling and multi-task perception through the linear complexity advantages of state-space models and task-specific designs. They not only break through the efficiency bottleneck of traditional Transformers but also provide precise and efficient solutions for temporal data processing in complex scenarios like autonomous driving, serving as a key technical bridge connecting sequence modeling and visual perception.

#### 4.3.3. Neural Architecture Search (NAS) in Multi-Task Learning

In the complex scenarios of autonomous driving, multi-task perception models must balance the demands of diverse tasks with in-vehicle computational constraints. Traditional manually designed architectures struggle to achieve the optimal synergy between performance and efficiency, making NAS a core solution through algorithmic automation. The core goal of NAS is to identify high-performance network structures that outperform manual designs with a data-driven approach. For the differentiated requirements of multi-task scenarios, such as object detection relying on multi-scale context, semantic segmentation emphasizing spatial details, and image classification requiring high-level semantic abstraction, NAS captures task commonalities and specificities through customized search spaces. For example, it automatically optimizes the stacking of convolutional layers, pooling strategies, and cross-layer connections in shared backbone networks to balance the generalization of low-level features and the task-specificity of high-level features, thereby selecting architectures with low parameter counts and excellent performance for resource-constrained in-vehicle platforms.

In single-task perception domains like object detection and semantic segmentation, NAS has significantly improved model efficiency and performance through tailored strategies. DAMO-YOLO [112] employs MAE-NAS to dynamically combine basic modules such as Res-blocks and integrate spatial pyramid pooling, balancing computational efficiency and detection accuracy under low-latency constraints. AutoDet [113] uses SA-NAS to construct a two-layer search space, automatically optimizing the feature pyramid network for object detection. Guided by detection AP, it balances efficiency and accuracy, reducing search costs by over 30 times compared to traditional methods and generating architectures that surpass a manually designed FPN. Vladimir Nekrasov et al. [114] dynamically generated decoder structures via an RNN controller, using a two-stage progressive search combined with auxiliary unit gradient optimization to create lightweight models that enable an efficient cross-task transfer for dense prediction tasks like semantic segmentation. Auto-DeepLab [115] constructs a hierarchical search space to jointly optimize network-level resolution transformations and unit-level operations. By leveraging differentiable relaxation, it breaks through the limitation of the traditional NAS, focusing solely on cell structures, efficiently discovering high-performance architectures suitable for semantic segmentation.

When migrating from single-task to multi-task scenarios, the design of the search space, cross-task knowledge transfer, and loss function optimization become key strategies. In terms of search space design, a shared-private hybrid architecture can be adopted, and MTNAS [116] is a typical example. MTNAS designs a two-stage NAS framework for multi-task learning in autonomous driving scenarios to optimize the overall network architecture. First, in the branch search stage, it optimizes the task-branch cell structure containing eight types of operations based on differentiable NAS technology, enabling the detection branch and segmentation branch to adapt to the requirements of multi-scale object localization and dense pixel prediction, respectively. Second, in the backbone search stage, it jointly optimizes the backbone architecture parameters and weights through pre-search on ImageNet and iteratively refines the cross-task shared features combined with multi-task loss. On the nuScenes [117] dataset, it achieves a 12% increase in the mAP of the multi-task model and a 40% reduction in computational load.

KTNAS [118] focuses on the issue of cross-task knowledge transfer efficiency and proposes an architecture embedding and transfer rank mechanism. It maps neural architectures to low-dimensional embedding vectors through node2vec to quantify architecture similarity and reduce search costs. It introduces the concept of transfer rank, builds a classifier based on historical data to screen high-priority architectures, and dynamically updates labels to alleviate negative transfer. During the evolution process, it combines tournament selection with crossover and mutation and supplements high-quality architectures from other tasks to the target population according to the transfer rank. Experiments on benchmarks such as NASBench-201 [119] verify that this method significantly improves the search efficiency and model performance in multi-task scenarios such as CIFAR-10/100 through efficient similarity representation and precise individual screening, proving the effectiveness of cross-task knowledge transfer.

In the collaborative design of loss functions and optimization objectives, TrajectoryNAS [120] constructs a multi-objective energy function that includes detection accuracy, prediction error, and inference latency. It dynamically adjusts the weights through a multi-objective simulated annealing algorithm and directly measures the latency on the target hardware to ensure real-time performance, solving the error accumulation problem of traditional cascading methods. On the NuScenes dataset, it achieves a 4.8% improvement in 3D trajectory prediction accuracy and a 1.1-fold reduction in latency.

In addition, the evolutionary multi-task convolutional neural architecture search framework proposed by Zhou et al. [121] combines evolutionary multi-task optimization with a low-fidelity evaluation strategy. This framework maintains independent populations for each task and sets up a shared archive to store high-quality cross-task architectures. Through an adaptive transfer strategy, it dynamically adjusts the frequency of knowledge transfer based on population diversity and knowledge helpfulness to reduce the impact of negative transfer. It uses a low-fidelity strategy during the evaluation to quickly screen the candidate architectures for cross-task migration and reduce unnecessary full-precision evaluations. In search spaces such as NAS201, this framework reduces the number of evaluations by 50–90%, achieving the collaborative advantages of evolutionary search and knowledge transfer in multi-task scenarios.

These studies collectively indicate that NAS provides a solution that balances accuracy, efficiency, and cross-task transferability for multi-task perception for autonomous driving through an automated architecture design and multi-dimensional optimization, promoting the practical deployment and application of intelligent systems in complex scenarios.

#### 4.3.4. AutoMTL: An Operator-Level Dynamic Sharing Framework for Multi-Task Learning

As a deepened application of NAS in multi-task learning (MTL), AutoMTL [122] addresses the pain point of traditional MTL relying on manual parameter design by constructing an operator-level dynamic sharing mechanism and an automated model generation pipeline.

The core mechanism of AutoMTL revolves around the fine-grained control of architectural design and the automation of the search process. First, the multi-task supermodel compiler transforms each network layer of any backbone CNN into configurable virtual computation nodes, allowing each task to independently choose to use shared backbone layers or task-specific layers or to skip the computation of that layer. This flexibility enables the adaptive adjustment of the model structure, so that low-level general features are preferentially shared to reduce parameter redundancy, while high-level task-specific features can be optimized independently to avoid interference. Second, the architecture search component employs differentiable policy optimization to automatically explore optimal sharing patterns. By integrating regularization to encourage low-level sharing for reduced memory usage and allowing a flexible high-level configuration according to task differences, it balances cross-task knowledge transfer and task-specific requirements. The entire process does not require a manual network architecture design. By simply inputting a backbone model and a task list, AutoMTL can automatically generate high-performance multi-task models through three stages: pretraining, policy optimization, and post-training. This framework supports any backbone networks, including ResNet and MobileNet, significantly reducing the development threshold. Its core advantage lies in the combination of fine-grained hierarchical sharing strategies and automated search. Through these approaches, the framework reduces the model parameters while improving the task accuracy by enabling the flexible configuration of layer-wise feature sharing at the operator level: low-level generic features are shared to minimize redundancy, and high-level task-specific features are optimized independently to avoid interference. This provides efficient and general solutions for multi-task scenarios such as autonomous driving and visual perception.

## 5. Evaluation

### 5.1. Datasets

In the field of multi-task perception for autonomous driving, various datasets are widely used. The BDD100K [104] dataset, created and maintained by the AI research laboratory at the University of California, Berkeley, and released in 2018, is one of the largest and most extensively applied driving video datasets. It is commonly utilized by numerous studies to evaluate the performance of image recognition algorithms in autonomous driving scenarios.

For the data collection, BDD100K employed cameras mounted on vehicles to gather data across multiple cities and surrounding areas in the United States. The dataset comprises 100,000 video clips, which are systematically divided into 70,000 for training, 10,000 for validation, and 20,000 for testing. These data encompass diverse driving scenarios and conditions such as different road types (urban roads and highways), various weather conditions (daytime, nighttime, rainy, and snowy), and distinct lighting conditions (bright sunlight, and overcast low light). Such diversity enables the comprehensive evaluation of image recognition algorithms in complex environments, enhancing the generalization capability of multi-task perception algorithms for real-world driving scenarios. Detailed information about BDD100K can be found in its original publication [104].

**Figure 4:**
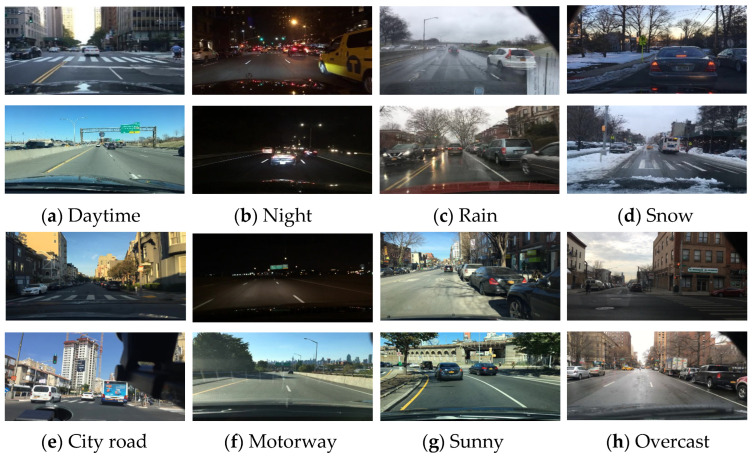
A partial image of the BDD100K dataset: (**a**) daytime; (**b**) night; (**c**) rain; (**d**) snow; (**e**) city road; (**f**) motorway; (**g**) sunny; and (**h**) overcast.

The dataset is designed with ten tasks. Among them, the object detection task has ten category labels to identify different types of targets such as vehicles, pedestrians, and traffic signs. The lane detection task has eight category labels to distinguish lane lines with different styles and attributes. The drivable area segmentation task has two category labels to define the safe driving areas for vehicles. A partial image of the BDD100K dataset is shown in Figure 4 below.

In addition to the BDD100K dataset, other commonly used datasets in autonomous driving multi-task perception research include KITTI [123], MSCOCO [106], and Cityscapes [124].

KITTI [123], co-created by the Karlsruhe Institute of Technology in Germany and Toyota Research Institute in the United States, collected data using vehicle-mounted LiDAR and cameras on urban and rural roads in Karlsruhe, Germany, covering weather conditions such as sunny and overcast days and traffic scenarios at different times of the day. The dataset provides 3D bounding box annotations for objects like vehicles and pedestrians, primarily supporting research on autonomous driving tasks such as object detection, semantic segmentation, and object tracking. Comprising 150,000 images with 11 label classes, KITTI includes 7481 images in the training set and 7518 images in the test set. In practical use, researchers often further partition the training set into training and validation subsets at a certain ratio (e.g., 8:2) to facilitate the algorithm training and evaluation. Details about KITTI can be retrieved from its original paper [123].

MSCOCO [106], a large-scale image dataset not specifically designed for autonomous driving, is applied to research on tasks like object detection relevant to autonomous driving. Collected across multiple global cities and regions, it encompasses diverse scenes such as urban streets and natural landscapes. The dataset provides annotations for tasks including object detection, instance segmentation, and image description, with 80 class labels. The MSCOCO 2014 dataset includes 82,783 images in the training set, 40,504 in the validation set, and 40,755 in the test set. The MSCOCO 2017 dataset contains 118,287 training images, 5000 validation images, and 40,670 test images. With its rich image categories and large data volume, MSCOCO supports research on the generality and generalization of algorithms for multi-task perception tasks like object detection. For in-depth information on MSCOCO, please refer to its original publication [106].

Cityscapes [124] is a large-scale dataset that specifically focuses on urban street scenes. It was compiled using vehicle-mounted cameras in multiple German cities. The dataset encompasses urban road scenarios under a wide range of weather and lighting conditions, such as sunny, overcast, and rainy days, along with daytime and nighttime settings. This rich collection of data offers targeted support for research on autonomous driving perception in urban environments. It plays a crucial role in enhancing the accuracy of algorithms for urban road segmentation and recognition. Regarding its annotations, Cityscapes provides meticulously labeled data for semantic segmentation tasks, with a total of 30 class labels. The dataset consists of 25,000 images distributed across different subsets. There are 2975 training video clips, each comprising 30 frames, 500 validation video clips, and 1525 test video clips. Information about Cityscapes can be found in its original reference [124].

The relevant information for these datasets is presented in Table 5.

The original papers of datasets like BDD100K, KITTI, MSCOCO, and Cityscapes play an important role. They explicitly document crucial details such as the data collection process, annotation specifics, and data splitting strategies. These details are fundamental for researchers to accurately understand and appropriately utilize these datasets, ensuring the reliability and reproducibility of the research results. For each of the mentioned datasets, such in-depth information can be found in their respective original publications. Additionally, when using these datasets for experiments, adjustments and configurations of the model parameters are critical factors influencing the algorithm performance. Due to differences in scenarios, annotation norms, and other aspects, distinct datasets are suitable for different model parameter settings. Researchers should elaborate on the process of model parameter adjustment in their papers to ensure experimental reproducibility and result reliability.

When creating new datasets, researchers are encouraged to share common information with the research community, including the design philosophy and application expectations behind the data, to enhance transparency and reproducibility and promote data sharing and research collaboration within the field. Such common information should include the following:

- Detailed data collection methods (sensors, environments, and conditions).
- Comprehensive annotation details (label types and guidelines).
- Clear information on data splits (training, validation, and testing).
- Ethical considerations.
- Accessibility and licensing information.

### 5.2. Evaluation Metrics

In deep learning, evaluation metrics compare the prediction results of a model with the ground truth values, measure the performance of the model numerically, and help to assess the model’s performance on a specific task. Different tasks such as classification, regression, and segmentation usually require different evaluation metrics. We combine classical methods with mainstream datasets to sort out the core evaluation metrics.

For object detection, Recall and mAP50 are commonly used for evaluation. The definitions of Recall and Precision are as follows:

(3) $$Recall=\frac{TP}{TP+FN}=\frac{TP}{P}$$

(4) $$Precision=\frac{TP}{TP+FP}$$

Recall refers to the probability that the samples that are actually positive are predicted as positive samples. Precision refers to the probability that among all the samples predicted as positive, the samples are actually positive. TP is the number of positive samples that are correctly classified as positive. TN is the number of negative samples that are correctly classified as negative. FP is the number of negative samples that are mistakenly classified as positive. FN is the number of positive samples that are mistakenly classified as negative. Recall and Precision are fundamental metrics for measuring the recall rate and precision rate of the detection models. For example, in the research of object detection in complex urban traffic scenarios, Faster R-CNN [10] evaluates the complete detection ability of the model for targets such as vehicles and pedestrians through Recall to avoid potential safety hazards caused by missed detections. Meanwhile, it controls the false detection rate using Precision to ensure the reliability of the detection results. Similarly, in experiments on the BDD100K dataset, YOLOv4 [16] improves the detection performance for small targets like traffic signs by optimizing the balance between Recall and Precision.

AP is the average precision. In object detection, a P-R curve is plotted with Recall as the abscissa and Precision as the ordinate, and the area under this curve is AP. The mAP is the average value of the APs of all the classes, and it is an important metric for evaluating the overall performance of the multi-class object detection models. The mAP50 measures the average precision of the model when the IoU threshold is 0.5.

The mAP is widely used as a comprehensive metric for multi-category detection for cross-dataset comparisons. For example, in a study of the COCO dataset [106], Mask R-CNN [85] comprehensively evaluates the model’s detection accuracy of 80 classes of targets by calculating the mAP of each class, which has become an important criterion to measure the generalization ability of the detection algorithm. The mAP50 is more practical in scenarios focusing on common overlap degrees, as YOLOP adopts mAP50 to evaluate the vehicle detection performance on the BDD100K dataset to ensure the model’s reliable accuracy in target localization on real roads.

Sometimes, the $F_{1}$ score is also used to evaluate the effect of traffic object detection. It is defined as the harmonic mean of Precision and Recall. The formula is as follows:

(5) $$F_{1}=2\cdot \frac{Precision\cdot Recall}{Precision+Recall}$$

**Figure 5:**
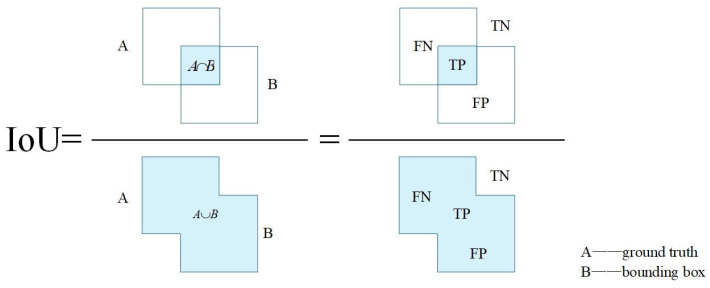
Schematic of IoU definition.

To evaluate the drivable area segmentation, the mIoU is commonly used. IoU, that is, the Intersection over Union, is used to measure the overlapping degree between the bounding box and the ground truth, as shown in Figure 5. The larger the ratio is, the higher the overlapping degree will be, and it is 1 when the bounding box and the ground truth completely overlap. The mIoU is calculated by taking the average of the IoUs of all the classes over all the samples.

The formulas for IoU and mIoU are as follows:

(6) $$IoU=\sum_{i=1}^{c}\frac{P_{nii}}{\sum_{j=1}^{c}P_{nij}+\sum_{j=1}^{c}P_{nji}-P_{nii}}$$

(7) $$mIoU=\frac{1}{N}\sum_{n=1}^{N}\left(\frac{1}{c}\sum_{i=1}^{c}\frac{P_{nii}}{\sum_{j=1}^{c}P_{nij}+\sum_{j=1}^{c}P_{nji}-P_{nii}}\right)$$

where c represents the total number of categories, N denotes the number of images in the validation set, $P_{nii}$ represents the number of pixels belonging to both the predicted and ground truth categories in the *n*-th sample, and $P_{nij}$ represents the number of pixels that are predicted as class j and simultaneously belong to class i in the *n*-th sample.

Lane segmentation is often evaluated using Accuracy and IoU. Accuracy refers to the percentage of correctly predicted results out of the total number of samples, as expressed below:

(8) $$Accuracy=\frac{TP+TN}{TP+TN+FP+FN}$$

## 6. Challenges and Opportunities

### 6.1. Challenges

In multi-task learning, the mutual influence among tasks is a key issue. There may be interference between different tasks, especially when dealing with complex scenarios, and such interference may significantly affect the overall performance of the system. Therefore, designing more efficient feature sharing and task branching strategies, making full use of the correlations among tasks, and reducing the interference between tasks remain urgent problems to be solved. At the same time, the problem of weight allocation between tasks is also a core challenge in multi-task learning. How to rationally design and optimize the weights of the loss functions to avoid the degradation of the performance of certain tasks and enable each task to achieve good performance jointly is a direction that requires in-depth research. In addition, it is necessary to carefully consider and select which parts the loss function of each subtask should include and which loss functions should be adopted, respectively.

In addition to the issues of the mutual influence between tasks and weight allocation, the problem of real-time performance is also an aspect that requires special attention in multi-task learning. Although multi-task methods are generally more efficient than single-task methods, when dealing with high-resolution images and complex tasks, a large amount of computational resources are still required. Therefore, how to further comprehensively optimize the network structure and improve the inference speed and accuracy will be the focus of future research. Furthermore, the lack of labeled data is also an important problem faced by multi-task learning. For multi-task learning, the acquisition and quality of the labeled data are of crucial importance. The diversity and complexity of autonomous driving scenarios make the collection of labeled data more difficult, especially for tasks such as depth estimation and semantic segmentation. Currently, there is a shortage of labeled data, and the efficiency of obtaining high-quality labeled data is low. Therefore, how to efficiently obtain high-quality labeled data is also an issue that needs to be considered in future research.

Nowadays, there is a certain degree of homogeneity and a lack of innovation in the design of network architectures and loss functions for multi-task perception for autonomous driving. Most of the network structures are based on convolutional neural networks. Although architectures based on Transformer have performed excellently in some fields, their applications in multi-task perception for autonomous driving are relatively scarce. Additionally, although emerging models such as Mamba have demonstrated great potential in long-sequence modeling and capturing long-range dependencies, their applications in multi-task perception for autonomous driving are still in the exploratory stage. In addition, the search process of NAS itself typically demands a substantial amount of computational resources and time, which may be impractical in real-world applications. In the context of autonomous driving scenarios, it is necessary to consider how to conduct architecture search efficiently with limited computational resources or explore the possibility of leveraging cloud computing resources for the search and then deploying the results to in-vehicle devices. When delving into these challenges and seeking opportunities for further research, readers should note that details regarding specific model parameters, training tuning processes, and other experimental specifics are not fully elaborated here. Researchers interested in such details are advised to refer to the original referenced literature and public code repositories cited in this paper to obtain the specific information, which can support more in-depth replication and extended research. It is hoped that this review will provide a valuable reference for industry practitioners and facilitate the practical application and innovative development of multi-task perception technologies for autonomous driving.

### 6.2. Opportunities

Multi-task perception technology for autonomous driving is booming. Its opportunities are closely linked to future development directions, showing broad application prospects. With the increasing complexity of various perception tasks in autonomous driving scenarios, such as object detection, lane detection, drivable area segmentation, and depth estimation, the importance of cross-task learning has become increasingly prominent. By strengthening the exploration of correlations and cooperation among different tasks to achieve information sharing, not only can the overall effect of multi-task learning be enhanced but also the perception ability for complex scenarios can be significantly improved. For example, object detection and semantic segmentation tasks can share the feature extraction network and improve the robustness and accuracy of the system through collaborative learning.

In the future, the development of multi-task perception technology for autonomous driving will place greater emphasis on the design of network architectures, continuing to explore emerging frameworks such as Transformer, Mamba, and NAS. These efforts are expected to bring more innovations and breakthroughs to multi-task perception for autonomous driving. Through technologies such as quantization and pruning, more lightweight multi-task learning networks will be designed to further improve computational efficiency. This will enable multi-task perception systems to operate more conveniently on actual vehicles and meet the stringent real-time requirements of autonomous driving. At the same time, by optimizing the network structure, adopting efficient inference engines, and combining with hardware acceleration technologies, the computational efficiency and accuracy of the system can be improved while ensuring real-time performance.

At the same time, improving the interpretability and security of the system is also an important direction for future research. By introducing model interpretation techniques and security mechanisms, it is possible to ensure that the decision-making process of the system is transparent and traceable and at the same time prevent security threats such as adversarial attacks and data tampering. The development of self-supervised learning and data augmentation techniques also provides the possibility of reducing the dependence on a large amount of labeled data. By using unlabeled image data for pretraining and then fine-tuning on a small amount of labeled data, the generalization ability of the model can be effectively improved, and the costs of data collection and annotation can be reduced simultaneously.

In addition, multi-modal fusion technology can be introduced, such as integrating information from other sensors like LiDAR and millimeter-wave radar, to make up for the deficiencies of a single visual sensor and further enhance the perception ability of the autonomous driving system.

In summary, multi-task perception technology for autonomous driving has broad development prospects in aspects such as cross-task learning, network architecture design, self-supervised learning, and multi-modal fusion. Through the continuous development and application of these technologies, autonomous driving systems will become more intelligent, efficient, and safe, laying a solid foundation for the future development of autonomous driving technology.

## 7. Conclusions

This paper presents a comprehensive review of multi-task perception technology for autonomous driving, focusing on vision-based tasks such as traffic object detection, drivable area segmentation, and lane detection. It explores the definition, function, and framework of multi-task learning; analyzes classic network architectures and loss function; and explores emerging frameworks for multi-task perception. It introduces the commonly used datasets and evaluation metrics. Finally, it discusses the current challenges and opportunities faced by multi-task perception.

## Figures and Tables

**Table 1:** Comparison of advantages and disadvantages of multi-task models.

Model	Advantages	Disadvantages
MultiNet [98]	1. Shared feature extractor, inference speed <45 ms 2. ROI alignment technique to improve detection accuracy 3. No need for explicit proposal generation network	1. Independent decoders which limit co-optimization of tasks 2. Fixed input size 3. Lack of multi-scale detection capability 4. Segmentation computing redundancy
DLT-Net [99]	1. Context tensor for cross-task feature fusion 2. High multi-task precision balance 3. Strong robustness in sparse scenarios	1. Limited performance on Jetson TX2 (<15 FPS) 2. Complex models and high consumption of training resources 3. Poor generalization on long-tailed data
YOLOP [82]	1. Real-time triple-task processing on Jetson TX2 (23 FPS) 2. Lightweight design for edge deployment 3. Adaptive cascade module optimizes segmentation	1. Delay introduced by multi-tasking module 2. Accuracy fluctuations in complex scenes 3. The need to trade off between accuracy and real time
A-YOLOM [101]	1. Ultra-lightweight architecture (<3 MB) 2. Adaptive multi-task weight allocation 3. Supporting dynamic resolution input	1. Limited performance on Jetson TX2 (<15 FPS) 2. Complex models and high consumption of training resources 3. Poor generalization on long-tailed data
HybridNets [83]	1. Hybrid CNN–Transformer architecture 2. Multi-modal fusion 3. End-to-end multi-task optimization	1. High computational requirements (>16 GB VRAM) 2. Difficult to deploy on edge devices 3. Slow training convergence

**Table 2:** Traffic object detection results.

Model	Recall (%)	mAP (%)	Speed (fps)	Image Size	Backbone+Neck
MultiNet [98]	81.3	60.2	8.6	224 × 224	VGG/Resnet
DLT-Net [99]	89.4	68.4	9.3	1280 × 720	VGG16 FPN
FasterR-CNN [10]	81.2	64.9	8.8	1000 × 600	VGG16/Resnet RPN
YOLOv5s	86.8	77.2	82	640 × 640	CSPDarknet53 FPN, PAN
YOLOv8n(det)	82.2	75.1	-	640 × 640	CSPDarknet FPN, PAN
YOLOP [82]	89.2	76.5	41	640 × 384	CSPDarknet SPP, FPN
A-YOLOM(n) [101]	85.3	78.0	-	640 × 640	CSPDarknet FPN, PAN
A-YOLOM(s) [101]	86.9	81.1	-	640 × 640	CSPDarknet FPN, PAN
HybridNets [83]	92.8	77.3	28	640 × 384	EfficientNet-B3 BiFPN
YOLOPv2 [103]	83.4	91.1	91	640 × 384	E-ELAN SPP, FPN

**Table 3:** Drivable area segmentation results.

Model	mIoU (%)	Speed (fps)	Image Size	Backbone+Neck
MultiNet [98]	71.6	8.6	224 × 224	VGG/Resnet
DLT-Net [99]	71.3	9.3	1280 × 720	VGG16 FPN
PSPNet [45]	89.6	11.1	473 × 473	ResNet PPM
YOLOv8n(seg)	78.1	-	640 × 640	CSPDarknet FPN, PAN
YOLOP [82]	91.6	41	640 × 384	CSPDarknet SPP, FPN
A-YOLOM(n) [101]	90.5	-	640 × 640	CSPDarknet FPN, PAN
A-YOLOM(s) [101]	91.0	-	640 × 640	CSPDarknet FPN, PAN
HybridNets [83]	90.5	28	640 × 384	EfficientNet-B3 BiFPN
YOLOPv2 [103]	93.2	91	640 × 384	E-ELAN SPP, FPN

**Table 4:** Lane detection results.

Model	Accuracy (%)	LaneLineIoU (fps)	Image Size	Backbone+Neck
Enet [53]	34.12	14.64	640 × 360	-
SCNN [72]	35.79	15.84	512 × 512	-
Enet-SAD [107]	36.56	16.02	360 × 640	-
YOLOP [82]	70.50	26.2	640 × 384	CSPDarknet SPP, FPN
HybridNets [83]	85.40	31.60	640 × 384	EfficientNet-B3 BiFPN
YOLOPv2 [103]	87.31	27.25	640 × 384	E-ELAN SPP, FPN

**Table 5:** Commonly used datasets.

Dataset	Year	Data Amount	Tag Categories	Scenarios
KITTI [123]	CVPR2012	150 K	11	urban and rural roads
MSCOCO [106]	ECCV2014	164 K	80	urban streets and natural landscapes
Cityscapes [124]	CVPR2016	25 K	30	urban street scenes
BDD100K [104]	CVPR2018	100 K	10/8/2	multiple cities and surrounding areas

Note: In the BDD100K dataset, “10/8/2” corresponds to the number of label classes for object detection, lane detection, and drivable area segmentation tasks, respectively.
